# LimoRhyde: A Flexible Approach for Differential Analysis of Rhythmic Transcriptome Data

**DOI:** 10.1177/0748730418813785

**Published:** 2018-11-25

**Authors:** Jordan M. Singer, Jacob J. Hughey

**Affiliations:** * Department of Biomedical Informatics, Vanderbilt University School of Medicine, Nashville, Tennessee; † Department of Biological Sciences, Vanderbilt University, Nashville, Tennessee

**Keywords:** differential expression, transcriptome data, cosinor, circadian rhythms, biological oscillations

## Abstract

Unraveling the effects of genetic or environmental perturbations on biological rhythms requires detecting changes in rhythmicity across multiple conditions. Although methods to detect rhythmicity in genome-scale data are well established, methods to detect changes in rhythmicity or changes in average expression between experimental conditions are often ad hoc and statistically unreliable. Here we present LimoRhyde (linear models for rhythmicity, design), a flexible approach for analyzing transcriptome data from circadian systems. Borrowing from cosinor regression, LimoRhyde decomposes circadian or zeitgeber time into multiple components to fit a linear model to the expression of each gene. The linear model can accommodate any number of additional experimental variables, whether discrete or continuous, making it straightforward to detect differential rhythmicity and differential expression using state-of-the-art methods for analyzing microarray and RNA-seq data. In this approach, differential rhythmicity corresponds to a statistical interaction between an experimental variable and circadian time, whereas differential expression corresponds to the main effect of an experimental variable while accounting for circadian time. To validate LimoRhyde’s performance, we applied it to simulated data. To demonstrate LimoRhyde’s versatility, we applied it to murine and human circadian transcriptome datasets acquired under various experimental designs. Our results show how LimoRhyde systematizes the analysis of such data, and suggest that LimoRhyde could prove valuable for assessing how circadian systems respond to perturbations.

In diverse species, from cyanobacteria to plants to mammals, circadian clocks drive rhythms in gene expression throughout the genome (Covington et al., 2008; Liu et al., 1995; Panda et al., 2002). Accordingly, transcriptome measurements have revealed circadian influences on physiology and potential applications for circadian medicine (Anafi et al., 2017; Hughey, 2017; Laing et al., 2017; Mure et al., 2018; Zhang et al., 2014). Transcriptome measurements are also beginning to reveal how circadian systems are affected by factors such as diet, infection, and cancer (Haspel et al., 2014; Masri et al., 2016; Tognini et al., 2017). Experiments probing these factors often give rise to datasets that include samples not just from multiple time points, but also from multiple conditions.

A common step in analyzing circadian or otherwise rhythmic transcriptome data is identifying which genes show evidence of rhythmic expression. This step can now be accomplished by various computational methods, including JTK_CYCLE and RAIN (Hughes et al., 2010; Hutchison et al., 2015; Thaben and Westermark, 2014). Importantly, though, these methods are only designed to detect rhythmic features (e.g., genes) based on samples from one condition. They are not designed to detect which features show a difference in rhythmicity between conditions (e.g., by comparing lists of rhythmic genes from each condition), and using them as such can lead to a high rate of false positives and false negatives (Thaben and Westermark, 2016). Indeed, the lack of a standard approach to analyze omics data from multiple conditions was highlighted in the recent guidelines for genome-scale analysis of biological rhythms (Hughes et al., 2017).

A classic approach for rhythm detection is cosinor regression (or harmonic regression), which is based on fitting a time series to the first harmonic of a Fourier series, i.e., sine and cosine curves of a set period (Cornelissen, 2014; Nelson et al., 1979). Because cosinor regression corresponds to a linear model, coefficients for even complex time series can be estimated efficiently using least squares. Inspired by cosinor regression, Thaben and Westermark recently made a significant advance in the statistically rigorous analysis of rhythmic transcriptome data from multiple conditions (Thaben and Westermark, 2016). Their method, called DODR, detects changes in rhythm amplitude, phase, and signal-to-noise ratio, which they call “differential rhythmicity.” DODR is relatively narrow in scope, though, as it is only designed to detect differential rhythmicity between 2 conditions. DODR cannot detect changes in average expression level between conditions, and cannot handle more complex experimental designs (e.g., with continuous variables, such as age).

In addition to being a fundamental part of cosinor regression, linear models are 1 of 2 components shared by nearly all state-of-the-art methods for assessing differential expression in transcriptome data. The second is called empirical Bayes. While linear models provide the ability to handle complex experimental designs, empirical Bayes shares information across genes to make more stable estimates of gene-wise variance and thereby improve statistical power and accuracy (Smyth, 2004). These methods can also appropriately deal with the non-Gaussian distributions of read counts from RNA-seq (Soneson and Delorenzi, 2013). Despite these methods’ flexibility and widespread success, their applications to circadian transcriptome data have been relatively limited (Hsu and Harmer, 2012; Montagner et al., 2016; Pembroke et al., 2015; Spörl et al., 2012). Encouragingly, recent work suggests that empirical Bayes can improve rhythmicity detection (Hutchison et al., 2018). To our knowledge, however, there has been no unification of cosinor-based approaches with these state-of-the-art tools for differential expression.

We sought to develop a general approach to systematically analyze circadian transcriptome data from various experimental designs. Our approach, which we call LimoRhyde (linear models for rhythmicity, design), builds on cosinor regression to express complex circadian experiments in terms of a linear model, which makes circadian transcriptome data amenable to analysis by existing tools for differential expression. We validated our approach in the 2-condition scenario by comparing it to DODR on simulated data and on 6 experimental datasets from mice. To explore LimoRhyde’s flexibility, we then applied it to 2 datasets from humans. Our results suggest that LimoRhyde offers a valuable framework for assessing how rhythmic biological systems respond to genetic and environmental perturbations.

## Materials and Methods

All data and code to reproduce this study are available at https://doi.org/10.6084/m9.figshare.5945569. The LimoRhyde R package is available at https://github.com/hugheylab/limorhyde.

### Processing the Gene Expression Data

For the RNA-seq datasets (GSE73552 and E-MTAB-3428), we downloaded the raw reads, then quantified gene-level abundances (based on Ensembl Gene IDs) in units of transcripts per million (TPM) using salmon v0.8.2 and tximport v1.6.0 (Patro et al., 2017; Soneson et al., 2015). We kept for analysis only those genes with TPM ⩾ 0.5 in at least half the samples. For all analyses and plots, we converted expression values to log_2_(TPM+1). For the microarray datasets, we downloaded the raw (Affymetrix) or processed (Agilent or Illumina) expression data from NCBI GEO, then used metapredict v0.0.0.9019 for mapping probes to Entrez Gene IDs, intra-study normalization, and log-transformation (Hughey and Butte, 2015). Details of all datasets are in Suppl. Table S1.

### Detecting Rhythmic, Differentially Rhythmic, and Differentially Expressed Genes using LimoRhyde and Limma

To make circadian transcriptome data amenable to analysis using linear models, LimoRhyde follows the strategy of cosinor regression, decomposing zeitgeber or circadian time into a sine and cosine of period 24 h. Although this decomposition is the simplest, one could also decompose time based on multiple harmonics of the Fourier series or on periodic splines. Thus, a single variable becomes at least 2 variables in the linear model. For data derived from several cycles in constant conditions, one could also include a linear time variable (e.g., time in free-run) to control for drift. Additional terms for condition, subject, or other covariates can be included as appropriate. In this approach, differential rhythmicity corresponds to a statistical interaction between the experimental factor of interest (e.g., genotype) and each term related to zeitgeber/circadian time. Differential expression, meanwhile, corresponds to the main effect of the experimental factor of interest.

For example, if the only variables of interest are zeitgeber time and genotype (with 2 values for the latter), then the linear model could be expressed as

$$\begin{array}{l} E(y_{gi})=\beta_{g0}+\beta_{g1}h_{i}+\beta_{g2}\;\cos\theta_{i}+\beta_{g3}\;\sin\theta_{i}\\ \;+\beta_{g4}h_{i}\cos\theta_{i}+\beta_{g5}h_{i}\sin\theta_{i}, \end{array}$$

where

$$\theta_{i}=\frac{2\pi t_{i}}{24},$$

$E\left(y_{gi}\right)$ is the expected (log-transformed) expression of gene *g* in sample *i*, $h_{i}$ is an indicator for sample *i*’s genotype (e.g., 0 for wild-type and 1 for knockout), $t_{i}$ is sample *i*’s zeitgeber or circadian time, and $\beta_{gj}$ are the unknown coefficients for gene *g*. The linear model could also be expressed in vector notation as

$$E(y_{gi})=x_{i}^{T}\beta_{g},$$

where

$$x_{i}=\left[1,h_{i},\cos\theta_{i},\sin\theta_{i},h_{i}\cos\theta_{i},h_{i}\sin\theta_{i}\right]$$

and $\beta_{g}$ is a column vector containing all the coefficients. In this example, differential rhythmicity corresponds to testing whether $\beta_{g4}$ and $\beta_{g5}$ are both equal to zero. Differential expression of non-differentially rhythmic genes, meanwhile, corresponds to removing the terms for $\beta_{g4}$ and $\beta_{g5}$, then testing whether $\beta_{g1}$ is equal to zero.

After constructing the linear model, the transcriptome data can be analyzed using multiple existing methods based on linear models and empirical Bayes. In this paper, we used limma v3.34.9 (Ritchie et al., 2015; Smyth, 2004). For all datasets (microarray and RNA-seq), we used limma with largely the default settings, except we allowed it to fit a mean-variance trend across genes (limma-trend) (Law et al., 2014). To control the false discovery rate, *p* values were converted to q-values using the method of Benjamini and Hochberg (1995).

In the datasets from mice, which have discrete time points spaced throughout the circadian cycle, we detected genes with rhythmic expression using RAIN (see next section). In the dataset based on samples from human brain (GSE71620), one experimental factor (age) is continuous and the zeitgeber time points are approximately randomly distributed. Therefore, to calculate a q-value of rhythmicity (accounting for age), we first used LimoRhyde to construct an additive model with terms for age, brain region, and zeitgeber time. The model does not include a term for donor because, although each donor has a corresponding sample from each of 2 brain regions, those 2 samples correspond to the same age and the same zeitgeber time, making it impossible to reliably account for inter-donor variation. We then used limma to perform a moderated F-test on the coefficients corresponding to the 2 terms for zeitgeber time.

### Detecting Rhythmic Genes using RAIN

For datasets with 2 conditions, our goal was to detect genes rhythmic in at least one condition. For datasets with discrete time points (all mouse datasets), we followed a similar procedure as used previously (Thaben and Westermark, 2016). We first ran RAIN v1.12.0 (default settings and period 24 h) separately on the samples from each condition, which resulted in a *p* value for each gene in each condition. We then used the minimum *p* value for each gene to calculate q-values of rhythmicity (q_rhy_). Comparing acrophase across conditions only makes sense if the gene is rhythmic in both conditions. We calculated q-values of being rhythmic in both conditions (q_rhy,max_) similarly, but using the maximum instead of the minimum *p* value.

### Detecting Rhythmic Genes using Lomb-Scargle

For the human brain dataset, we also attempted to identify rhythmic genes using the implementation of Lomb-Scargle in the MetaCycle R package v1.1.0 (Wu et al., 2016). We first adjusted the expression values for age and brain region, then ran Lomb-Scargle on the residual expression values from all samples. We tested only one value for the period (24 h).

### Detecting Differentially Rhythmic Genes using DODR

We used the DODR R package v0.99.2 (Thaben and Westermark, 2016), specifically the robustDODR function, which tests for a combination of amplitude and phase change and corresponds to the moderated F-test in limma. By default, DODR first divides each gene’s measurements by the mean for that gene in that condition. In our simulations, however, the expression of genes was centered around zero. For generalizability and for consistency between our analyses of experimental and simulated data, we instead subtracted from each measurement the mean for that gene in that condition, then called robustDODR with division-by-mean normalization disabled.

### Comparing LimoRhyde and DODR for Detecting Differential Rhythmicity

To validate LimoRhyde’s ability to detect differential rhythmicity between 2 conditions, we created simulations with non-rhythmic, rhythmic, and differentially rhythmic genes. The simulated data were based on drawing 2 samples every 2 h for 24 h from 2 conditions, and were designed to roughly mimic the properties of real data. Each simulated dataset contained 10,080 genes, with 75% of genes non-rhythmic and 25% rhythmic, and 25% of the rhythmic genes differentially rhythmic (i.e., having a difference in amplitude and/or phase between conditions). Rhythmic expression was modeled as a sinusoid with a period of 24 h. Noise in expression was modeled as additive Gaussian with a standard deviation of 1.

For each simulated dataset, we used LimoRhyde (followed by limma) and robustDODR to calculate the *p* value of differential rhythmicity for each gene. Based on those *p* values, we then used the precrec R package v0.9.1 to calculate a receiver operating characteristic (ROC) curve for distinguishing each set of differentially rhythmic genes from the genes that were rhythmic but not differentially rhythmic. By using the known labels (a benefit of the simulations), this analysis avoided confounding with the method for detecting rhythmic genes and focused on the best-case performance of LimoRhyde and DODR for detecting differential rhythmicity.

To evaluate the agreement between LimoRhyde and DODR in calling genes differentially rhythmic on experimental data, we calculated Cohen’s kappa at various q-value cutoffs using the irr R package v0.84. To estimate each method’s tendency to call false positives in each dataset, we first identified genes rhythmic in at least one condition using RAIN on the true sample labels, then permuted the sample labels (wild-type or knockout) within samples from the same time point. This strategy attempts to preserve rhythmic expression patterns, but remove differential rhythmicity. For each dataset, we then calculated the mean number of differentially rhythmic genes (across 50 permutations) at various q-value cutoffs. Because the number of differentially rhythmic genes varies substantially across datasets, we summarized the overall results using the geometric mean.

To ensure a sufficient number of rhythmic genes for comparison, we used a cutoff of q_rhy_ ⩽ 0.1 for 5 of the 6 datasets. We used a cutoff of q_rhy_ ⩽ 0.15 for E-MTAB-3428, which has only 4 time points and 8 samples per genotype.

### Calculating Gene-wise Rhythmic Parameters using ZeitZeiger

We estimated rhythm amplitude and zeitgeber/circadian time of peak expression (acrophase) using ZeitZeiger v1.0.0.5 with default settings (Hughey et al., 2016; Hughey and Butte, 2016). For the mouse datasets, we ran ZeitZeiger separately on the samples from each condition. For the human brain dataset, to calculate each gene’s overall rhythmic properties, we used LimoRhyde and limma to adjust the expression values for age and brain region, then ran ZeitZeiger on the residual expression values from all samples. To estimate the change in rhythmic properties with age, we split donors by age into younger 50% and older 50% groups, then calculated the rhythm amplitude and acrophase on the unadjusted expression values within each cohort. For all datasets, we calculated Δamplitude as the arithmetic difference in rhythm amplitude, and Δacrophase as the circular difference (constrained between −12 h and +12 h).

### Performing Gene Set Analysis using CAMERA

To identify gene sets enriched for differential expression, we used the camera function in the limma R package (Wu and Smyth, 2012). CAMERA takes as input an expression matrix, a list of gene sets, a design matrix corresponding to a linear model, and a single contrast (e.g., genotype or age). As in the limma analysis, we allowed CAMERA to fit a mean-variance trend. To identify gene sets enriched for differential rhythm amplitude, we used the cameraPR function (default settings), which takes as input a vector of gene-wise statistics (in our case, Δamplitude) and a list of gene sets. We used the mouse and human C5 GO (gene ontology) gene sets of MSigDB v5.2 (Liberzon et al., 2011), which are available at http://bioinf.wehi.edu.au/software/MSigDB/index.html. The gene sets are based on Entrez Gene IDs, so for the gene set analysis of GSE73552, we mapped Ensembl Gene IDs to Entrez Gene IDs using the org.Mm.eg.db R package, keeping only genes with a one-to-one mapping.

## Results

### Applying LimoRhyde to Circadian Transcriptome Data from a Two-Condition Design

To develop a workflow for using LimoRhyde, we first sought to analyze a circadian transcriptome dataset that is representative of a common experimental design, in which samples are acquired at discrete time points throughout the circadian cycle in 2 conditions. We selected a dataset that included samples taken every 4 h from livers of wild-type and clock gene knockout Arntl^^-/-^^ mice under night-restricted feeding in LD 12-12, with gene expression measured by RNA-seq (Atger et al., 2015). Starting with the RNA-seq reads, we estimated gene-level abundances using Salmon and tximport (Patro et al., 2017; Soneson et al., 2015). Using RAIN (Thaben and Westermark, 2014), we then identified 2434 genes rhythmic in at least one genotype (q_rhy_ ⩽ 0.01; Table 1 and Suppl. Fig. S1A).

**Table 1.** Workflow Used to Detect Rhythmic, Differentially Rhythmic, and Differentially Expressed Genes in Circadian Transcriptome Data from Livers of Wild-type and Arntl^-/-^ Mice under Night-restricted Feeding (GSE73552).

Analysis Step	Genes Tested	Method	Variables in Linear Model
1. Rhythmicity	All	RAIN	-
2. Diff. rhythmicity	Rhythmic	LimoRhyde	$\left[1,h_{i},\cos\theta_{i},\sin\theta_{i},h_{i}\cos\cdot \theta_{i},h_{i}\sin\cdot \theta_{i}\right]$
3. Diff. expression	Non-diff. rhythmic	LimoRhyde	$\left[1,h_{i},\cos\theta_{i},\sin\theta_{i}\right]$

In the vectors of variables, $h_{i}$ and $\theta_{i}$ correspond to genotype (encoded as an indicator variable) and zeitgeber time (in radians) for sample *i*, respectively. Bold font indicates the variables whose corresponding coefficients were of interest at each step. Arrows indicate that rhythmic genes were tested for differential rhythmicity, and non-differentially rhythmic genes were tested for differential expression.

We next used LimoRhyde to express the experimental design in terms of a linear model (Table 1) and used limma to determine which rhythmic genes showed evidence of differential rhythmicity between wild-type and Arntl^-/-^ mice. Limma is a general method for analyzing microarray and RNA-seq data based on linear models and empirical Bayes (Ritchie et al., 2015; Smyth, 2004). We used limma to calculate a moderated F-statistic for each rhythmic gene, which tests the null hypothesis that both coefficients corresponding to the interaction between genotype and zeitgeber time are zero. This amounts to testing for a difference in a combination of rhythm amplitude and phase (Thaben and Westermark, 2016). Of 2434 rhythmic genes, 1641 genes were differentially rhythmic at a cutoff of q_DR_ ⩽ 0.1 (Suppl. Fig. S1B). Of the 16 genes with the lowest q_DR_, 8 genes are part of or directly driven by the core circadian clock (Rorc, Arntl, Nr1d1, Dbp, Cry1, Ciart, Nr1d2, and Per3).

**Figure 1.**
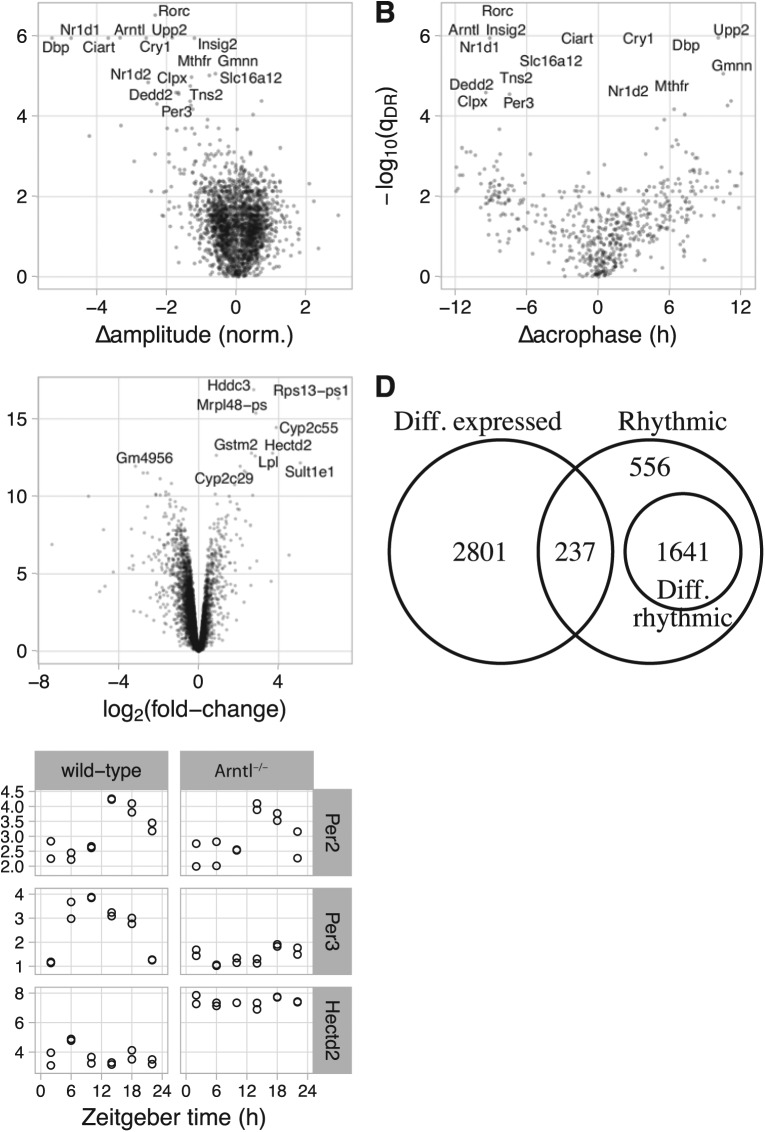
Using LimoRhyde to analyze circadian transcriptome data from livers of wild-type and Arntl^^-/-^^ mice under night-restricted feeding (GSE73552). (A) Scatterplot of -log_10_(q_DR_) vs. Δamplitude. q_DR_ corresponds to a rhythmic gene’s q-value of differential rhythmicity. Δamplitude corresponds to the change in rhythm amplitude between genotypes, where a negative value indicates lower amplitude in Arntl^-/-^. In A-C, each point represents a gene. In A and B, the 16 rhythmic genes with the highest -log_10_(q_DR_) are labeled. (B) Scatterplot of -log_10_(q_DR_) vs. Δacrophase. The latter corresponds to the change in zeitgeber time of peak expression, where a positive value indicates a phase advance in Arntl^-/-^. Only genes with q-value for rhythmicity in both genotypes ⩽ 0.2 are shown. (C) Scatterplot of -log_10_(q_DE_) vs. log_2_ (fold-change). q_DE_ corresponds to the q-value of differential expression. A positive log_2_ (fold-change) indicates higher average expression in Arntl^-/-^. The 10 genes with the highest -log_10_(q_DE_) are labeled. (D) Venn diagram of genes meeting criteria for rhythmicity (q-value of rhythmicity q_rhy_ ⩽ 0.01), differential rhythmicity (q_DR_ ⩽ 0.1), and differential expression (q_DE_ ⩽ 0.1). (E) Plots of 3 example genes. Each point represents a sample. Based on the criteria, Per2 is classified as rhythmic only, Per3 as differentially rhythmic, and Hectd2 as differentially expressed only.

Although the moderated F-statistic can provide evidence of a change in rhythmicity, it does not indicate the nature of the change. Therefore, for each rhythmic gene, we used ZeitZeiger (Hughey et al., 2016) to quantify the rhythm amplitude and the zeitgeber time of peak expression (acrophase) in wild-type and Arntl^-/-^ mice. We found that the genes with the best evidence for differential rhythmicity had strongly reduced rhythm amplitude in Arntl^-/-^ mice (Fig. 1A). Among genes that exhibited at least moderate evidence of rhythmicity in each genotype (q_rhy,max_ ⩽ 0.2; see Materials and Methods), changes in acrophase were widely distributed (Fig. 1B). As expected (Thaben and Westermark, 2016), genes with stronger evidence of differential rhythmicity tended to have larger absolute changes in acrophase.

We then used a simpler linear model, one lacking an interaction between genotype and zeitgeber time, to identify genes differentially expressed between wild-type and Arntl^-/-^ mice. Here differential expression refers to a difference in average expression level between genotypes, accounting for possible rhythmicity. For this step, we considered only the 11,737 genes for which there was not strong evidence of differential rhythmicity (q_DR_ > 0.1) or for which differential rhythmicity was not examined (q_rhy_ > 0.01). Among these genes, 3038 genes were differentially expressed (q_DE_ ⩽ 0.01), of which 301 genes had an absolute log_2_ fold-change >1 (Fig. 1C).

We also investigated how the numbers of genes classified as differentially rhythmic and differentially expressed were affected by the criteria used at each step. We found that the number of differentially rhythmic genes increased as the cutoffs for rhythmicity and differential rhythmicity became less stringent (Suppl. Fig. S2A). The number of differentially expressed genes, on the other hand, increased as the cutoff for differential rhythmicity became more stringent (to the extent this led to a decrease in the number of differentially rhythmic genes) and as the cutoff for differential expression became less stringent (Suppl. Fig. S2B).

Finally, to complement the gene-wise analysis, we used a method called CAMERA to perform gene set analysis (Wu and Smyth, 2012). Because methods such as CAMERA are unable to work directly with the F-statistics of differential rhythmicity (which have only a positive sign), we instead plugged into CAMERA the differences in rhythm amplitude as quantified by ZeitZeiger. Consistent with the gene-wise analysis, 4 of the 5 top-ranked gene sets with altered rhythm amplitude were related to circadian rhythms (all with q ⩽ 10^−8^ and reduced amplitude in Arntl^-/-^; Suppl. Table S2). Gene sets enriched for differential expression, meanwhile, tended to be related to the ribosome and various catabolic processes (with increased expression in Arntl^-/-^; Suppl. Table S3).

Given criteria for rhythmicity, differential rhythmicity, and differential expression, the assignment of genes to each group can be expressed as a Venn diagram (Fig. 1D). To illustrate the various expression patterns, we show 3 genes as examples (Fig. 1E). Taken together, these results suggest that LimoRhyde provides a cohesive framework for the differential analysis of circadian transcriptome data.

### Comparing LimoRhyde and DODR in the Assessment of Differential Rhythmicity

Detecting differential rhythmicity between 2 conditions is the use case for DODR. As in LimoRhyde, differential rhythmicity in DODR is defined as a statistical interaction in a linear model based on cosinor regression. Although DODR does not use empirical Bayes to share information between genes, it does use rank-based statistics to achieve robustness to outlier samples.

To validate LimoRhyde’s ability to detect differential rhythmicity between 2 conditions, and to benchmark its performance against that of DODR, we simulated transcriptome datasets containing non-rhythmic, rhythmic, and differentially rhythmic genes (see Materials and Methods). The latter were defined by values for mean amplitude (relative to the standard deviation of additive Gaussian noise), difference in amplitude, and difference in phase (example time-course in Suppl. Fig. S3A).

**Figure 2.**
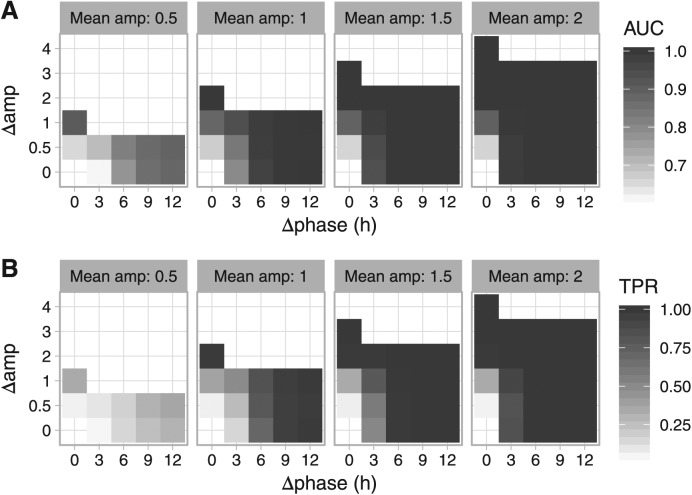
Evaluating the detection of differential rhythmicity in simulated data with LimoRhyde. Simulations were based on drawing 2 samples every 2 h for 24 h from 2 conditions. Each simulated dataset contained 10,080 genes, with 75% of genes non-rhythmic, 25% rhythmic, and 25% of the rhythmic genes differentially rhythmic between the 2 conditions. Each set of differentially rhythmic genes was defined by mean amplitude, difference in amplitude (Δamp) and difference in phase (Δphase). In total, we created 56 simulated datasets, giving 504 genes in each set of differentially rhythmic genes. Difference in amplitude could equal up to twice the mean amplitude, at which point the gene was non-rhythmic in one condition, and thus the difference in phase was undefined. The additive Gaussian noise in expression values had a standard deviation of 1. Using the *p* values of differential rhythmicity from LimoRhyde (followed by limma), we calculated a receiver operating characteristic (ROC) curve for distinguishing each set of differentially rhythmic genes from all genes that were rhythmic but not differentially rhythmic. Heatmaps of (A) area under the ROC curve (AUC) and (B) fraction of differentially rhythmic genes having a nominal *p* value of differential rhythmicity ⩽ 0.01 (true positive rate, TPR).

For each simulated dataset, we used LimoRhyde and DODR to calculate the *p* value of differential rhythmicity (p_DR_) for each gene. We first verified that p_DR_ for non-differentially rhythmic genes was uniformly distributed between zero and one, as expected under the null hypothesis (Suppl. Fig. S3B). We then used p_DR_ from each method to calculate the area under the ROC curve (AUC) for distinguishing each set of differentially rhythmic genes from all genes that were rhythmic but not differentially rhythmic. As the AUC is not based on any one threshold, we also calculated the fraction of differentially rhythmic genes for which p_DR_ ⩽ 0.01 (true positive rate, TPR, not adjusted for multiple testing). Values of AUC and TPR for both LimoRhyde and DODR approached one (perfect detection) as the difference in amplitude or phase between conditions increased. (Fig. 2 shows values for LimoRhyde; Suppl. Fig. S4 shows the small differences between the 2 methods.) These results indicate that, independent of the method used to detect rhythmicity, both methods provide similarly strong detection of differential rhythmicity.

To compare LimoRhyde and DODR on experimental data, we assembled 6 circadian transcriptome datasets (4 microarray, 2 RNA-seq). Each dataset included samples taken at discrete circadian time points from wild-type mice and clock gene knockout mice (Suppl. Table S1). For each dataset, we used RAIN to detect genes rhythmic in at least one genotype, applying a less stringent cutoff (q_rhy_ ⩽ 0.1) to have more genes for comparison. For rhythmic genes, we then used LimoRhyde and DODR to calculate q-values of differential rhythmicity (Fig. 2A). The median runtimes per dataset were 0.3 s for LimoRhyde and limma and 41 s for DODR.

**Figure 3.**
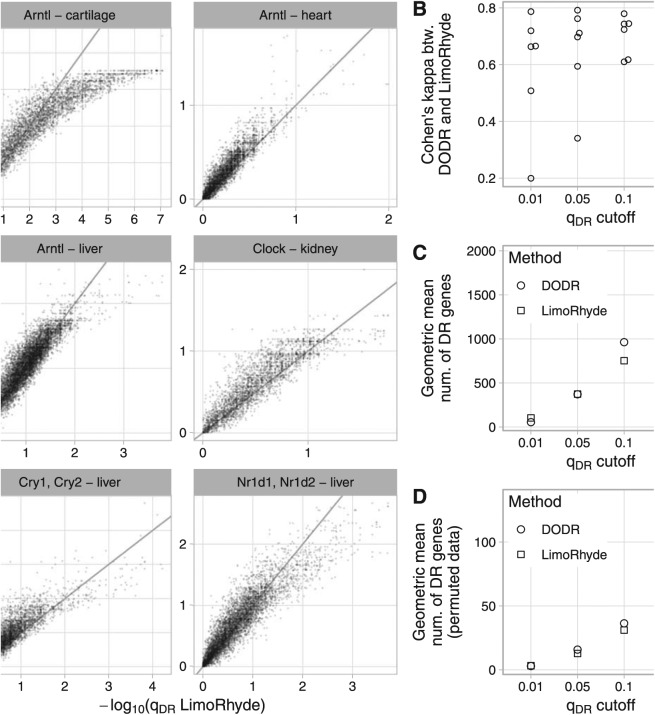
Comparing LimoRhyde (followed by limma) and DODR for detecting differential rhythmicity (DR) between wild-type and clock gene knockout mice. For details of datasets, see Suppl. Table S1. In each dataset, rhythmic genes were identified using RAIN. (A) Scatterplots of q-value of differentially rhythmicity as calculated by each method. The title of each plot indicates the knocked-out gene(s) and the tissue in which gene expression was measured. Each point represents a rhythmic gene. The line indicates y = x. For each dataset, up to 15 genes with extremely high -log_10_(q_DR_ LimoRhyde) are not shown. (B) Cohen’s kappa, a measure of inter-rater agreement, between DODR and LimoRhyde at various q-value cutoffs. Each point represents a dataset. (C) Geometric mean (across datasets) of the number of differentially rhythmic genes at various q-value cutoffs. (D) Geometric mean (across datasets) of the mean (across permutations) number of differentially rhythmic genes at various q_DR_ cutoffs, in data in which the sample labels (wild-type or knockout) were permuted. Labels were permuted after identifying rhythmic genes, and were only permuted within samples at the same time point. Thus, DR genes identified in permuted data can be considered false positives for differential rhythmicity.

Overall, q-values from the 2 methods were highly correlated (median Spearman correlation coefficient 0.91). In addition, based on Cohen’s kappa, LimoRhyde and DODR showed moderate to strong agreement at various q-value cutoffs (Fig. 3B). Although the number of differentially rhythmic genes varied between datasets, LimoRhyde tended to select slightly more genes than DODR at a low q-value cutoff (q_DR_ ⩽ 0.01) and somewhat fewer genes at higher q-value cutoffs (q_DR_ ⩽ 0.1 or q_DR_ ⩽ 0.2; Fig. 3C and Suppl. Fig. S5A). To evaluate the ability of the 2 methods to control false positives, we performed permutation testing on each dataset (see Materials and Methods). Both methods effectively controlled false positives, detecting many fewer differentially rhythmic genes on permuted data than on the unpermuted data; although, again, LimoRhyde tended to select fewer genes (i.e., was more conservative) than DODR at higher q-value cutoffs (Fig. 3D and Suppl. Fig. S5B). These results suggest that LimoRhyde (followed by limma) and DODR provide comparable detection of differential rhythmicity in circadian transcriptome data.

### Applying LimoRhyde to Human Transcriptome Data from Diverse Experimental Designs

We next explored the flexibility of LimoRhyde using 2 transcriptome datasets from humans, each of which has a different experimental design than the datasets from mice. The first dataset from humans was based on brain tissue from postmortem donors, with the zeitgeber time for each sample based on the respective donor’s time of death, date of death, and geographic location (Chen et al., 2016). The 146 donors ranged in age from 16 to 96 years old (M ± SD, 50.7 ± 15.3). Given how sleep-wake patterns change with age (Roenneberg et al., 2004; Yoon et al., 2003), this dataset presents an excellent opportunity to examine the interaction between aging and circadian rhythms in 2 regions of the human prefrontal cortex (Brodmann’s areas 11 and 47). The original analysis, however, was forced to discretize donors into younger and older, which discards information and sacrifices statistical power. LimoRhyde, on the other hand, can accommodate continuous variables such as age without discretizing them.

**Figure 4.**
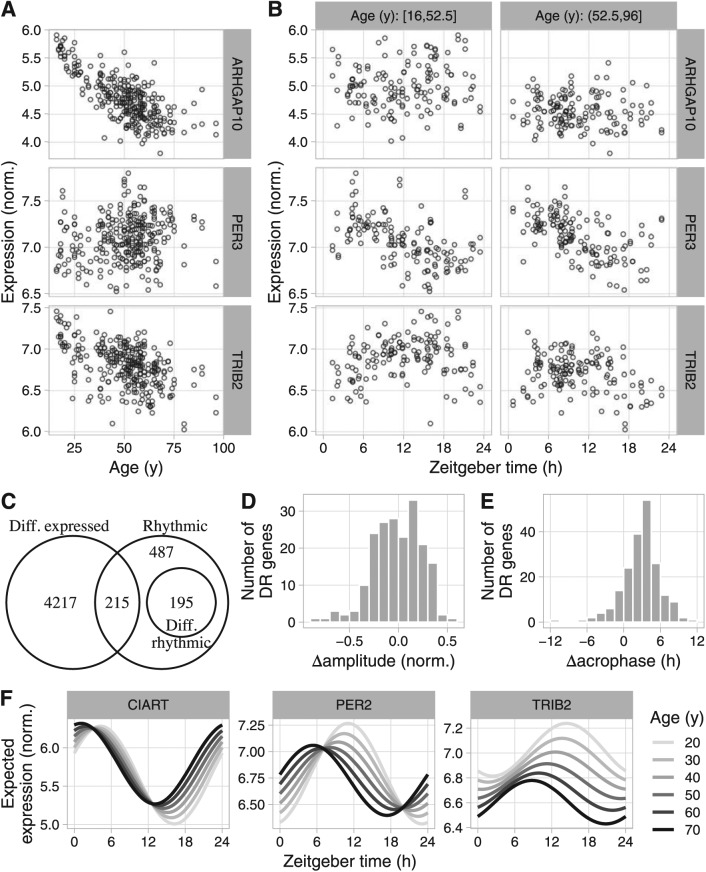
Using LimoRhyde to analyze transcriptome data based on postmortem samples from human brain (GSE71620). (A) and (B) Scatterplots for 3 example genes, showing log-normalized expression as a function of age and zeitgeber time of death within younger and older donors. Each point represents a sample. ARHGAP10 is classified as differentially expressed only, PER3 as rhythmic only, and TRIB2 as differentially rhythmic. (C) Venn diagram of genes meeting criteria for rhythmicity (q_rhy_ ⩽ 0.1 and rhythm amplitude ⩾ 0.1), differential rhythmicity (q_DR_ ⩽ 0.1), and differential expression (q_DE_ ⩽ 0.01). (D) and (E) Histograms of Δamplitude and Δacrophase for differentially rhythmic genes between younger and older donors, calculated using ZeitZeiger. Positive Δamplitude indicates higher rhythm amplitude in older donors. Positive Δacrophase indicates a phase advance in older donors. (F) Expected expression, based on linear model coefficients, as a function of zeitgeber time and age for 3 of the most strongly differentially rhythmic genes (by q_DR_).

Because the time points are based on times of death, they are approximately randomly spaced, making use of RAIN or JTK_CYCLE infeasible. Therefore, to identify rhythmic genes, we used an additive model in LimoRhyde, including terms for age (as a continuous variable in years), zeitgeber time, and brain region (Table 2; 3 example genes are shown in Fig. 4A-B). This additive model is equivalent to cosinor regression. To estimate each gene’s overall rhythm amplitude, we applied ZeitZeiger to the residuals of an additive model lacking terms for zeitgeber time (see Materials and Methods). Applying the criteria of q_rhy_ ⩽ 0.1 and rhythm amplitude ⩾ 0.1, we identified 891 genes as rhythmic (Fig. 4C and Suppl. Fig. S6A). In contrast, the Lomb-Scargle method, which can also handle randomly spaced time-points (Glynn et al., 2006), identified only 30 genes as rhythmic (q ⩽ 0.1; Suppl. Fig. S6B).

**Table 2.** Workflow Used to Detect Rhythmic, Differentially Rhythmic, and Differentially Expressed Genes in Postmortem Samples from Human brain (GSE71620).

Analysis step	Genes tested	Method	Variables in linear model
1. Rhythmicity	All	LimoRhyde	$\left[1,r_{i},a_{i},\cos\cdot \theta_{i},\sin\cdot \theta_{i}\right]$
2. Diff. rhythmicity	Rhythmic	LimoRhyde	$\left[1,r_{i},a_{i},\cos\theta_{i},\sin\theta_{i},a_{i}\cos\cdot \theta_{i},a_{i}\sin\cdot \theta_{i}\right]$
3. Diff. expression	Non-diff. rhythmic	LimoRhyde	$\left[1,r_{i},a_{i},\cos\theta_{i},\sin\theta_{i}\right]$

In the vectors of variables, $r_{i}$, $a_{i}$, and $\theta_{i}$ correspond to brain region (encoded as an indicator variable), age (in years, as a continuous variable), and zeitgeber time (in radians) for sample *i*, respectively. Bold font indicates the variables whose corresponding coefficients were of interest at each step. Arrows indicate that rhythmic genes were tested for differential rhythmicity, and non-differentially rhythmic genes were tested for differential expression.

To find genes whose rhythmic expression varied with age, we altered the linear model to include an interaction between age and zeitgeber time, maintaining age as a continuous variable (Table 2). Of the 891 genes that met our criteria for rhythmicity, 195 genes were differentially rhythmic (q_DR_ ⩽ 0.1; Fig. 4C and Suppl. Fig. S6C). To estimate the age-dependent changes in rhythm amplitude and acrophase of differentially rhythmic genes, we applied ZeitZeiger to samples from the younger 50% and older 50% of donors. Changes in rhythm amplitude were centered near zero, with similar numbers of genes showing increased or decreased amplitude in older donors (Fig. 4D). Genes with decreased rhythm amplitude were enriched for involvement in leukocyte-mediated immunity and the adaptive immune response (Suppl. Table S5). Changes in acrophase were shifted from zero, corresponding to a mean advance of 3.1 h in older donors (circular mean; Fig. 4E). Finally, to visualize differential rhythmicity of individual genes, we used the linear model coefficients to calculate expected expression as a function of zeitgeber time and age (Fig. 4F).

Using the original additive model (Table 1), we then identified 4551 genes whose expression increased or decreased with age, accounting for zeitgeber time (q_DE_ ⩽ 0.01; Fig. 4C and Suppl. Fig. S6D). The numbers of differentially rhythmic and differentially expressed genes showed a similar dependence on the criteria for rhythmicity, differential rhythmicity, and differential expression as in the mouse liver dataset (Suppl. Fig. S7). Genes whose expression decreased with age were strongly enriched for involvement in glutamate receptor signaling, synapse structure and activity, and mitochondria (Suppl. Table S4), which is consistent with previous findings (Lu et al., 2004).

The second dataset from humans was based on suction-blister epidermis samples acquired from 20 subjects at 3 time points (0930 h, 1430 h, and 1930 h) (Spörl et al., 2012). The original analysis, which used limma but considered the time points as categorical variables (ANOVA) and did not adjust for inter-subject variation, identified 294 genes whose expression varied with time of day (q ⩽ 0.05).

To analyze the dataset using LimoRhyde, we constructed a linear model with terms for subject and time of day (Suppl. Fig. S8A). We then used limma to perform a moderated F-test on the 2 coefficients corresponding to time of day, which identified 1436 genes with time-of-day-dependent expression (q ⩽ 0.05; Suppl. Fig. S8B). Among the 15 top-ranked genes were 8 core clock genes (NR1D1, PER3, CIART, NPAS2, PER1, ARNTL, NR1D1, and PER2, all with q ⩽ 2×10^−8^).

Because this dataset has exactly 3 time points, the LimoRhyde time decomposition and ANOVA are equivalent; they both correspond to 2 parameters in the linear model (the increased number of detected genes in our analysis is a result of adjusting for inter-subject variation). As the number of time points increases, though, LimoRhyde will continue to favor genes whose expression varies sinusoidally over time, whereas ANOVA, which ignores the relationship between time points, will not. Taken together, these examples demonstrate how LimoRhyde enables a statistically rigorous analysis of circadian transcriptome data from diverse experimental designs.

## Discussion

Despite the increasing complexity of experiments to interrogate rhythmic biological systems, methods to fully analyze the resulting genome-scale data have remained largely ad hoc. Here we described LimoRhyde, a unified approach to detect gene-wise differential rhythmicity and differential expression in circadian or otherwise rhythmic transcriptome data. LimoRhyde is inspired by cosinor regression and is applicable to data from any experimental design that can be described by a linear model. LimoRhyde thus functions as an adapter, making circadian transcriptome data amenable to analysis by the ever-improving and growing set of methods designed for the differential analysis of microarray and RNA-seq data.

For detecting differential rhythmicity in the common 2-condition scenario, our results suggest that LimoRhyde performs comparably to DODR. Although LimoRhyde (followed by limma) is considerably faster, the absolute difference in runtime is negligible compared to the amount of time required to perform the experiments.

LimoRhyde distinguishes itself from DODR by its versatility. First, LimoRhyde can be used to detect rhythmic or time-of-day–dependent gene expression in datasets in which time points are either randomly spaced or do not cover the full circadian cycle, scenarios for which the current implementations of methods such as JTK_CYCLE and RAIN are ill-suited. In this application, LimoRhyde is conceptually equivalent to cosinor regression, with the advantage of using empirical Bayes procedures in methods such as limma to share information between genes. Second, LimoRhyde enables the detection of differential expression between conditions, accounting for possible rhythmicity. This could reveal expression changes in genes whose mRNAs are too stable to be rhythmic or differentially rhythmic (Lück et al., 2014). Our results suggest that a typical circadian experiment is well powered to detect even relatively small log fold-changes. Third, LimoRhyde can be applied to transcriptome data from complex experimental designs. Here we analyzed a dataset in which an experimental variable was continuous, and a dataset in which multiple samples were collected from each participant.

While LimoRhyde provides rigorous gene-wise *p* values, other methods are useful for interpretation. Given a set of differentially rhythmic genes, methods such as ZeitZeiger can quantify the changes in rhythm amplitude and phase. Although such post hoc comparisons are not statistical tests, it may be possible in the future to test specifically for a difference in one quantity or the other. Furthermore, gene set analysis methods such as CAMERA can identify biological processes that are enriched for changes in average expression level or in rhythm amplitude. An analogous method called Phase Set Enrichment Analysis could identify processes enriched for changes in phase (Zhang et al., 2016).

Regardless of the computational method, detecting differential rhythmicity and differential expression requires 2 assumptions. The first is a value for the period of the rhythm. For typical experiments using entrained or newly free-running organisms, the assumed period will likely correspond to the period of the most recent zeitgeber (T) or possibly the free-running period of the organism (tau). Neither DODR nor LimoRhyde are currently designed to detect differences in period. The second assumption is an alignment of all time points, whether from conditions with different values of tau (if free-running) or different photoperiods, to one scale. For example, if the photoperiod varied between conditions, the results will depend on whether time zero in each photoperiod is defined as the time of lights on or the time of lights off. Consequently, we advise caution when calculating and interpreting differential rhythmicity in datasets based on free-running organisms for which tau varies considerably between conditions. The danger of this experimental design is that, if the time points are not aligned properly, the results will be confounded by differences in the organisms’ intrinsic circadian phase (Hsu and Harmer, 2012).

In addition to these assumptions, categorizing genes as rhythmic, differentially rhythmic, and differentially expressed—although convenient—requires arbitrary cutoffs of q-value and/or rhythm amplitude. An alternative approach would be to test the coefficients for the main effect and the statistical interaction jointly, which would identify genes showing evidence for either differential rhythmicity or differential expression.

Multiple features of LimoRhyde remain to be explored. For example, although we used LimoRhyde in conjunction with limma, which is fast and can handle both microarray and RNA-seq data, LimoRhyde is compatible with multiple other methods for differential expression analysis. In addition, although here we decomposed time using sine and cosine curves (as in cosinor), it is also possible to apply a decomposition based on a periodic smoothing spline (as in ZeitZeiger). LimoRhyde could also be used to detect differences in higher-order harmonics of circadian gene expression (Hughes et al., 2009).

In conclusion, we have developed a general approach for the differential analysis of rhythmic transcriptome data. We concentrated on microarray and RNA-seq data, but given limma’s success on proteomics, DNA methylation, and ChIP-Seq data (Brusniak et al., 2008; Lun and Smyth, 2014; Maksimovic et al., 2012), we are optimistic that LimoRhyde could be applied to other types of genome-scale data as well. Altogether, LimoRhyde can help ensure that our ability to analyze rhythmic omics data continues to scale with our ability to acquire it.

## Supplemental Material

limorhyde_supp – Supplemental material for LimoRhyde: A Flexible Approach for Differential Analysis of Rhythmic Transcriptome Data Click here for additional data file. Supplemental material, limorhyde_supp for LimoRhyde: A Flexible Approach for Differential Analysis of Rhythmic Transcriptome Data by Jordan M. Singer and Jacob J. Hughey in Journal of Biological Rhythms

## Supplemental Material

Table_S1 – Supplemental material for LimoRhyde: A Flexible Approach for Differential Analysis of Rhythmic Transcriptome Data Click here for additional data file. Supplemental material, Table_S1 for LimoRhyde: A Flexible Approach for Differential Analysis of Rhythmic Transcriptome Data by Jordan M. Singer and Jacob J. Hughey in Journal of Biological Rhythms

## Supplemental Material

Table_S2 – Supplemental material for LimoRhyde: A Flexible Approach for Differential Analysis of Rhythmic Transcriptome Data Click here for additional data file. Supplemental material, Table_S2 for LimoRhyde: A Flexible Approach for Differential Analysis of Rhythmic Transcriptome Data by Jordan M. Singer and Jacob J. Hughey in Journal of Biological Rhythms

## Supplemental Material

Table_S3 – Supplemental material for LimoRhyde: A Flexible Approach for Differential Analysis of Rhythmic Transcriptome Data Click here for additional data file. Supplemental material, Table_S3 for LimoRhyde: A Flexible Approach for Differential Analysis of Rhythmic Transcriptome Data by Jordan M. Singer and Jacob J. Hughey in Journal of Biological Rhythms

## Supplemental Material

Table_S4 – Supplemental material for LimoRhyde: A Flexible Approach for Differential Analysis of Rhythmic Transcriptome Data Click here for additional data file. Supplemental material, Table_S4 for LimoRhyde: A Flexible Approach for Differential Analysis of Rhythmic Transcriptome Data by Jordan M. Singer and Jacob J. Hughey in Journal of Biological Rhythms

## Supplemental Material

Table_S5 – Supplemental material for LimoRhyde: A Flexible Approach for Differential Analysis of Rhythmic Transcriptome Data Click here for additional data file. Supplemental material, Table_S5 for LimoRhyde: A Flexible Approach for Differential Analysis of Rhythmic Transcriptome Data by Jordan M. Singer and Jacob J. Hughey in Journal of Biological Rhythms
