# Supplementary material for: LimoRhyde: A Flexible Approach for Differential Analysis of Rhythmic Transcriptome Data
Source: J Biol Rhythms. 2018 Nov 25;34(1):5–18. doi: 10.1177/0748730418813785 (PMC6376636; doi:10.1177/0748730418813785)
Supplement: limorhyde_supp – Supplemental material for LimoRhyde: A Flexible Approach for Differential Analysis of Rhythmic Transcriptome Data [file limorhyde_supp.pdf]

Supplemental Figures for:

LimoRhyde: a flexible approach for differential analysis of rhythmic transcriptome data

Jordan M. Singer[1] and Jacob J. Hughey[1,2,*]

[1]Department of Biomedical Informatics, Vanderbilt University School of Medicine, Nashville, Tennessee; [2]Department of Biological Sciences, Vanderbilt University, Nashville, Tennessee

*To whom all correspondence should be addressed: jakejhughey@gmail.com

Figure S1

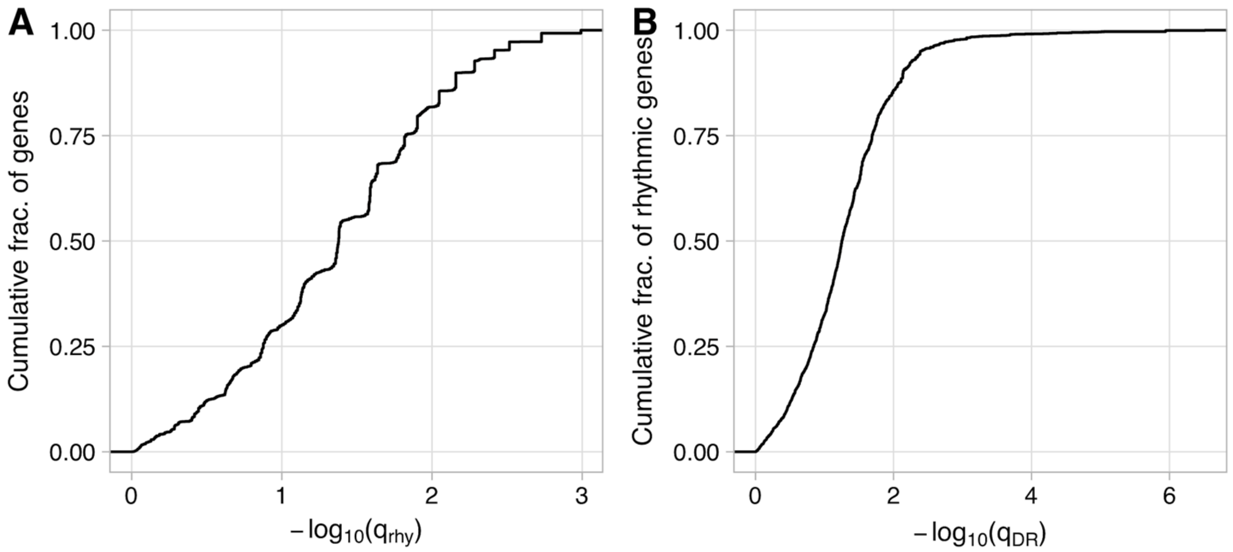

Analyzing circadian transcriptome data from liver of wild-type and Arntl-/- mice under night-restricted feeding (GSE73552). **(A)** Empirical cumulative distribution function for $-\log_{10}(q_{rhy})$, where q_{rhy} corresponds to a gene's q-value of being rhythmic in at least one genotype, calculated using RAIN. **(B)** Empirical cumulative distribution function for $-\log_{10}(q_{DR})$, where q_{DR} corresponds to a rhythmic gene's q-value of differential rhythmicity, calculated using LimoRhyde and limma and based only on genes for which $q_{rhy} \leq 0.01$.

Figure S2

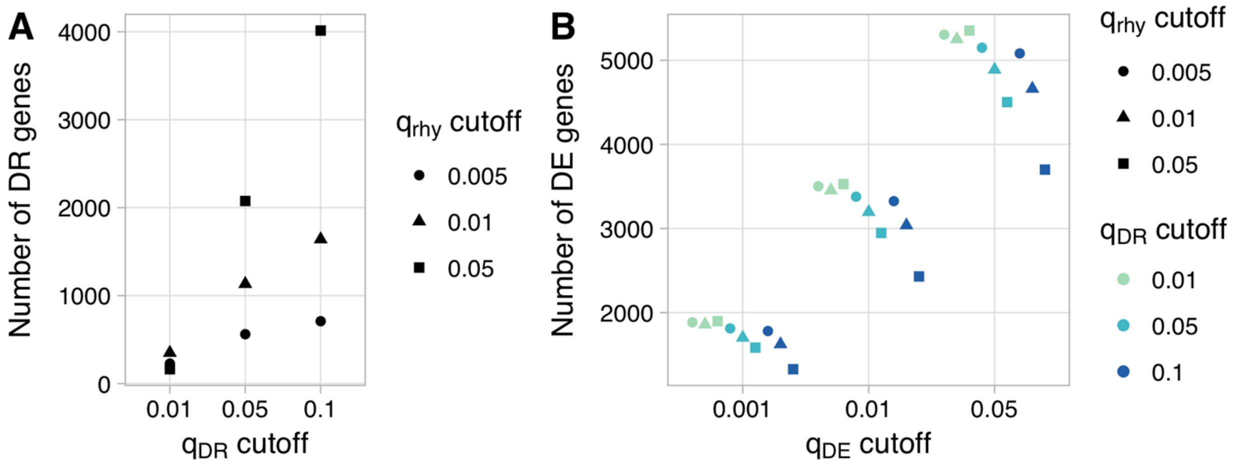

Analyzing the number of differentially rhythmic (DR) and differentially expressed (DE) genes in mouse liver (GSE73552) under various q-value cutoffs for rhythmicity (q_{rhy}), differential rhythmicity (q_{DR}), and differential expression (q_{DE}). **(A)** Number of DR genes as a function of cutoffs for q_{rhy} and q_{DR}. **(B)** Number of DE genes as a function of cutoffs for q_{rhy}, q_{DR}, and q_{DE}.

Figure S3

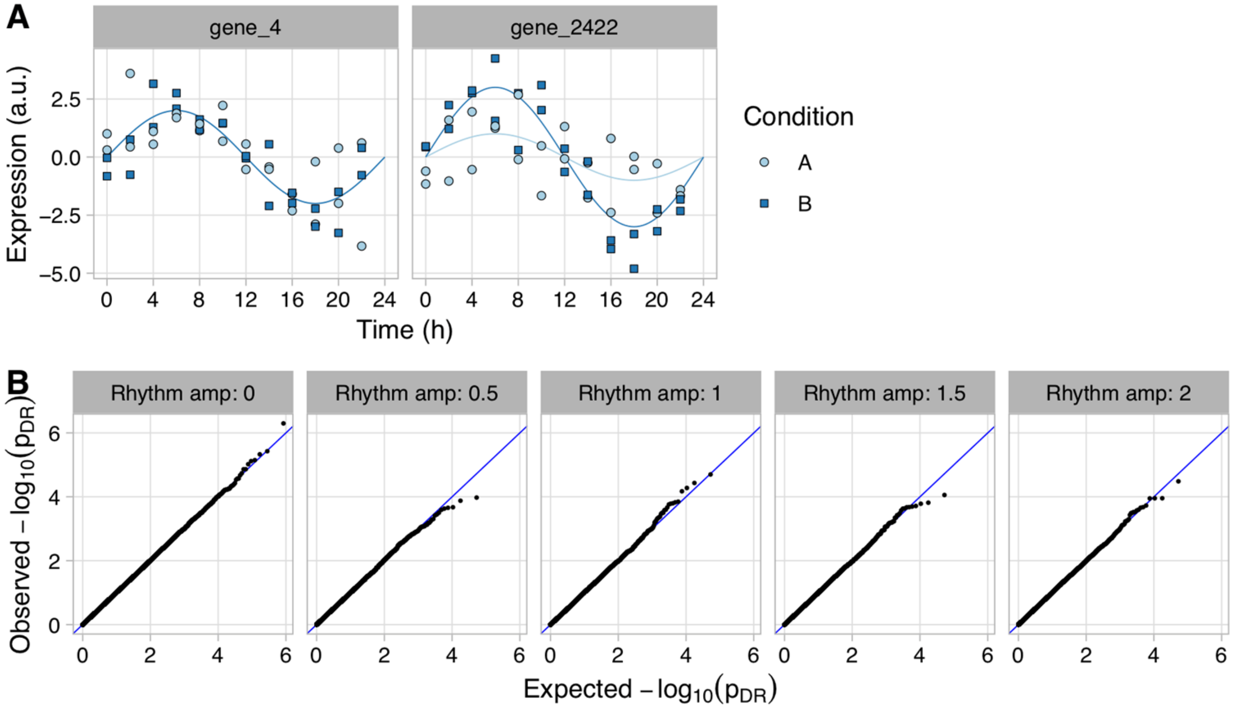

Validating LimoRhyde using simulations. Plots are based on the same simulated data shown in Fig. 2. **(A)** Scatterplots of the expression time-course and the underlying sine curves for two simulated genes. Gene_4 is identically rhythmic in both conditions (amplitude of 2). Gene_2422 is differentially rhythmic, with amplitude 1 in condition A and amplitude 3 in condition B. **(B)** Quantile-quantile plots of p-value of differential rhythmicity (p_{DR}) for non-differentially rhythmic genes. Amplitude of zero corresponds to non-rhythmic genes (n = 423,360). Amplitudes greater than zero correspond to genes identically rhythmic in both conditions (n = 26,488 per value of rhythm amplitude). The p-values are uniformly distributed between 0 and 1, as expected under the null hypothesis.

Figure S4

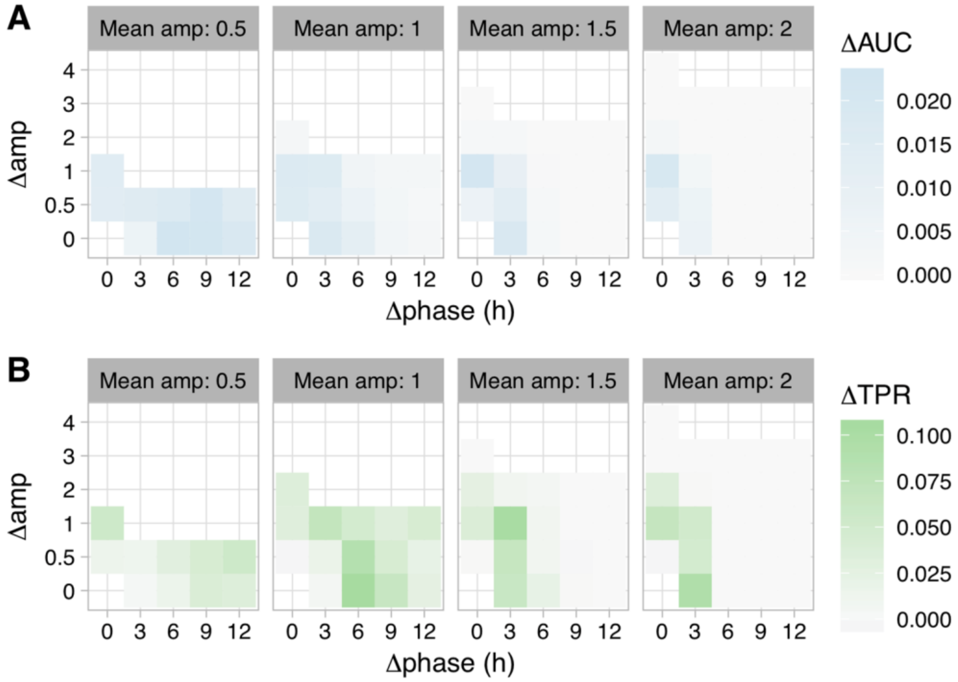

Comparing LimoRhyde and DODR on simulated data. We ran the robustDODR test on the same data used to make Fig. 2. Heatmaps of the differences in **(A)** area under the ROC curve (ΔAUC) and **(B)** fraction of differentially rhythmic genes having nominal p-value of differential rhythmicity ≤ 0.01 (ΔTPR). Positive values indicate higher AUC or TPR for LimoRhyde, respectively.

Figure S5

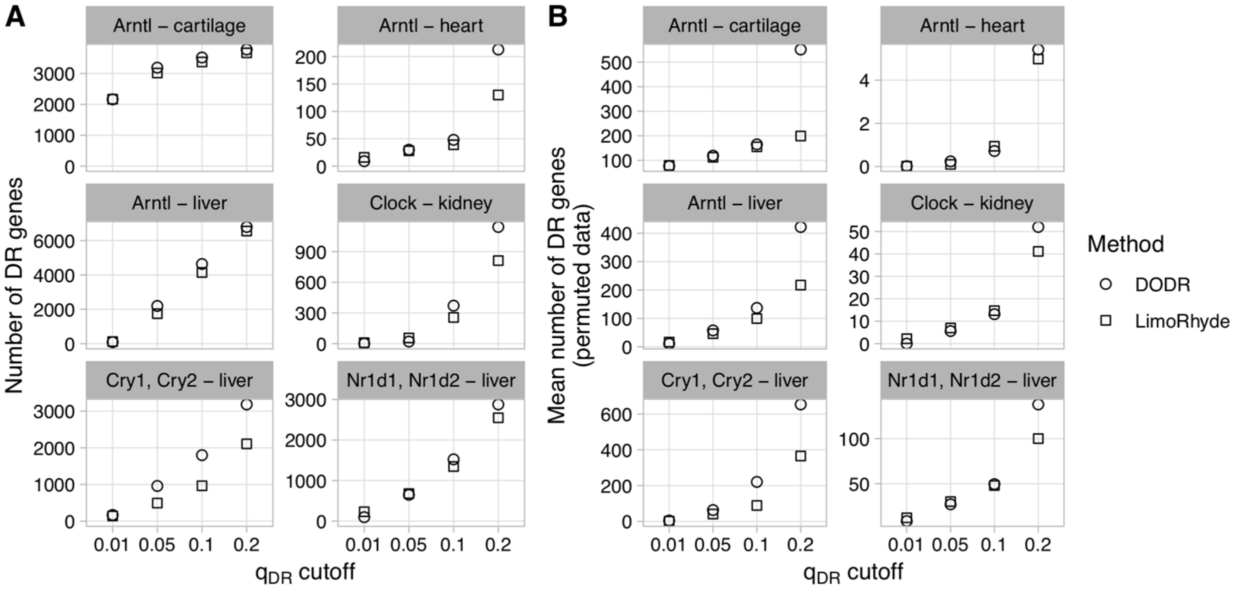

Comparing LimoRhyde (followed by limma) and DODR for detecting differential rhythmicity between wild-type and clock gene knockout mice. The title of each plot indicates the knocked-out gene(s) and the tissue in which gene expression was measured. For details of datasets, see Suppl. Table S1. In each dataset, rhythmic genes were identified using RAIN ($q_{rhy} \leq 0.1$). **(A)** Number of differentially rhythmic (DR) genes at various q-value cutoffs. **(B)** Number of differentially rhythmic genes at various q-value cutoffs, in data in which the sample labels (wild-type or knockout) were permuted. Labels were permuted after identifying rhythmic genes, and were only permuted within samples at the same time-point. Thus, DR genes identified in permuted data can be considered false positives for differential rhythmicity.

Figure S6

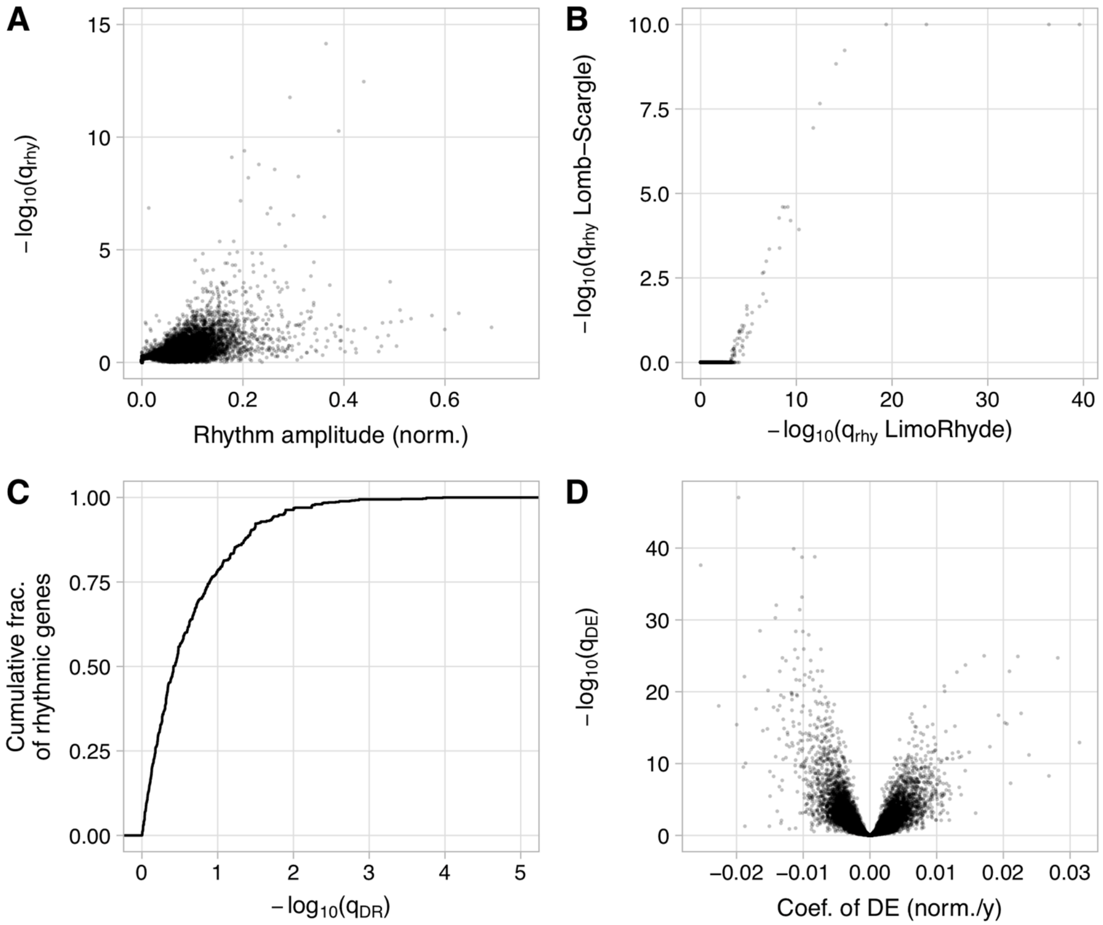

Analyzing circadian transcriptome data based on postmortem samples from human brain (GSE71620). **(A)** Scatterplot of -$\log_{10}(q_{rhy})$ vs. rhythm amplitude, where q_{rhy} is the q-value of rhythmicity. Each point represents a gene. q_{rhy} was calculated using LimoRhyde and limma with an additive model including terms for zeitgeber time, age, and brain region. Rhythm amplitude was calculated using ZeitZeiger and the residuals of an additive model including terms for age and brain region. Rhythm amplitude is in log-normalized units of expression. **(B)** Comparison of q-values for rhythmicity between LimoRhyde and Lomb-Scargle. Each point represents a gene. The q-values for four genes according to Lomb-Scargle were zero, and these q-values were set to 10^{-10}. **(C)** Empirical cumulative distribution function of -$\log_{10}(q_{DR})$, where q_{DR} is the q-value of differential rhythmicity, based on a linear model with an interaction age and zeitgeber time, considering only genes having $q_{rhy} \leq 0.1$ and rhythm amplitude ≥ 0.1. **(D)** Scatterplot of -$\log_{10}(q_{DE})$ vs. the coefficient of differential expression, where q_{DE} is the q-value of differential expression. Each point represents a gene. Because age is continuous, the coefficient does not correspond to a log fold-change.

Figure S7

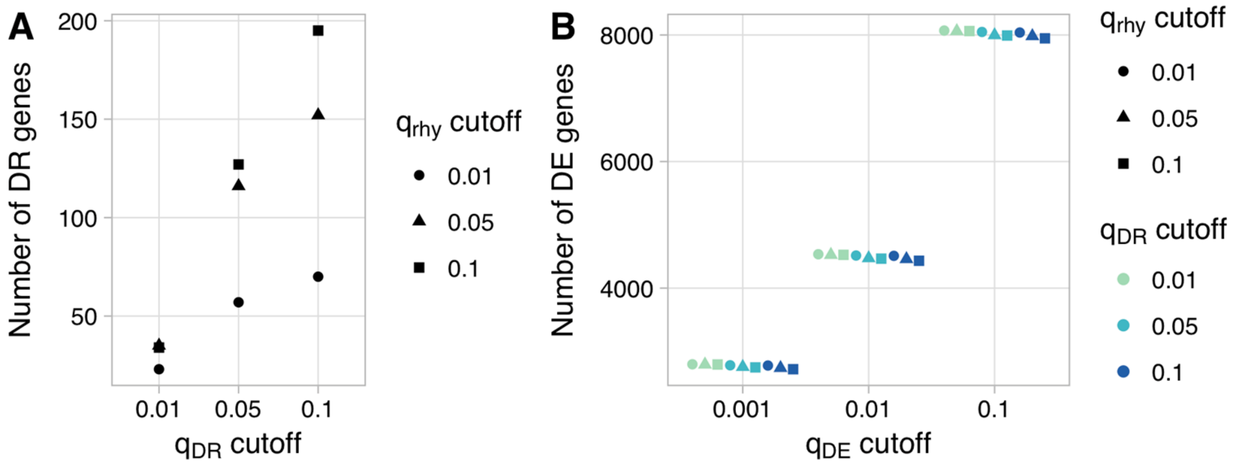

Analyzing the number of differentially rhythmic (DR) and differentially expressed (DE) genes in human brain (GSE71620) under various q-value cutoffs for rhythmicity (q_{rhy}), differential rhythmicity (q_{DR}), and differential expression (q_{DE}). **(A)** Number of DR genes as a function of cutoffs for q_{rhy} and q_{DR}. **(B)** Number of DE genes as a function of cutoffs for q_{rhy}, q_{DR}, and q_{DE}.

Figure S8

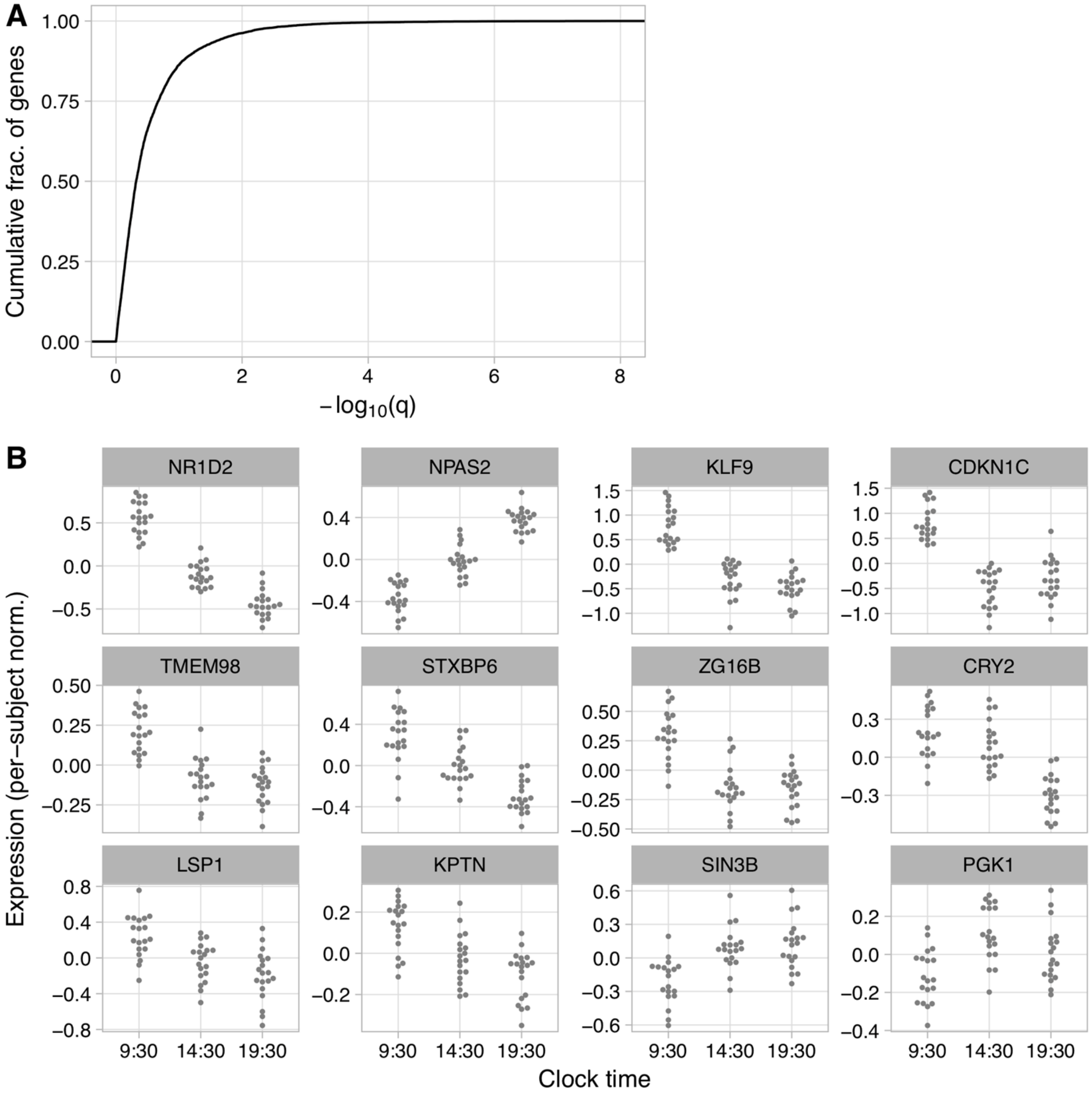

Using LimoRhyde identify genes whose expression varies with time of day in human epidermis (GSE35635). The variables used in the linear model were $[1, s_i, \mathbf{\cos\theta_i}, \mathbf{\sin\theta_i}]$, where bold font indicates the variables of interest. $s_i$and $\theta_i$correspond to subject (encoded as indicator variables) and zeitgeber time (in radians) for sample i, respectively. **(A)** Empirical cumulative distribution function of q-value of time-of-day-dependent expression. Fourteen genes with $q \leq 10^{-8}$ are not shown. **(B)** Per-subject normalized expression for 12 example genes. Each point represents a sample. Expression values correspond to the residuals of a limma fit based only on subject. Genes in the top row have $q \leq 10^{-8}$, genes in the middle row have $10^{-6} < q \leq 10^{-4}$, and genes in the bottom row have $10^{-3} < q \leq 0.1$.
